# Efficacy of a Feed Dispenser for Horses in Decreasing Cribbing Behaviour

**DOI:** 10.1155/2016/4698602

**Published:** 2016-10-13

**Authors:** Silvia Mazzola, Clara Palestrini, Simona Cannas, Eleonora Fè, Gaia Lisa Bagnato, Daniele Vigo, Diane Frank, Michela Minero

**Affiliations:** ^1^Dipartimento di Medicina Veterinaria, Università degli Studi di Milano, Via Celoria 10, 20133 Milan, Italy; ^2^Faculté de Médecine Vétérinaire, Département de Sciences Cliniques, Université de Montréal, No. 3200, rue Sicotte, Saint-Hyacinthe, QC, Canada J2S 2M2

## Abstract

Cribbing is an oral stereotypy, tends to develop in captive animals as a means to cope with stress, and may be indicative of reduced welfare. Highly energetic diets ingested in a short time are one of the most relevant risk factors for the development of cribbing. The aim of this study was to verify whether feeding cribbing horses through a dispenser that delivers small quantities of concentrate when activated by the animal decreases cribbing behaviour, modifies feeding behaviour, or induces frustration. Ten horses (mean age 14 y), balanced for sex, breed, and size (mean height 162 cm), were divided into two groups of 5 horses each:* Cribbing* and* Control*. Animals were trained to use the dispenser and videorecorded continuously for 15 consecutive days from 1 h prior to feeding to 2 h after feeding in order to measure their behaviours. The feed dispenser, Quaryka®, induced an increase in time necessary to finish the ration in both groups of horses (*P* < 0.05). With Quaryka, cribbers showed a significant reduction of time spent cribbing (*P* < 0.05). After removal of the feed dispenser (Post-Quaryka), cribbing behaviour significantly increased. The use of Quaryka may be particularly beneficial in horses fed high-energy diets and ingesting the food too quickly.

## 1. Introduction

Stereotypies are defined as invariant and repetitive behaviour patterns that seem to have no function [1]. They are reported in more than 15% of domesticated horses [2] and are known as the disease of domestication [3], since they have never been observed in free-ranging feral horses. They may be indicative of reduced welfare [4, 5] but it is not self-evident whether stereotypies are representative of the current situation or of a previous suboptimal condition. This is based on the findings that once a stereotypic behaviour is established, it will become a habit, and it is difficult to stop or rectify it [6–8]. Cribbing horses may be stressed more easily than unaffected horses [9, 10]. It has been shown that attempts to inhibit this behaviour through the use of anticribbing collars or other physical devices may significantly impact equine welfare, by reducing the horse's ability to cope with stress without addressing the underlying cause [11].

Epidemiological and experimental studies provide quite an accurate understanding of the prevalence, underlying mechanisms, and owner perceptions of cribbing behaviour. These studies have shown that many factors can be associated with an increased risk of cribbing, including management conditions that prevent foraging opportunities and social contact, provision of high concentrate diets, and abrupt weaning [12]. Since cribbers perform the behaviour most frequently following delivery of concentrated feed, it has been suggested that diet may be implicated [13]. As a consequence of high-energy carbohydrate feed administration, cribbing horses ingest food quickly and have a lower production of saliva, a higher gastric fermentability [14], and acid fermentation in the cecum and large intestine [13]. The latter phenomenon may be related to a higher transit time of feed in the large intestine, indicating that the orocecal digestion in cribbing horses is less efficient compared to healthy subjects. The action of cribbing could therefore be an attempt to stimulate the receptors of the oral tissues to increase the flow of saliva and thus buffer the acidity of the gastrointestinal tract [15]. Equine behaviour and welfare scientists agree that management of cribbing horses should focus on improvement of life conditions and feeding management rather than on attempts at physical prevention of the behaviour.

The aim of this study was to verify whether feeding cribbing horses through a dispenser that delivers small quantities of concentrate when activated by the animal decreases or eliminates cribbing behaviour, modifies feeding behaviour, or induces frustration. This study describes the effect of a feed dispenser, Quaryka, on feeding time budget of cribbing horses.

## 2. Research Methods

Ten horses, balanced for breed and sex, aged between six and 20 years (mean age: 14 years), were recruited. All subjects were deemed healthy following a physical examination and were exempt of medical treatment, except for vaccination and deworming.

Horses were divided into two groups: five* Cribbing horses* and five* Control horses* (Table 2). Cribbing horses had been stereotyping for at least one month and had never been treated for the condition whereas Control horses had never exhibited the stereotypy. All subjects were kept under the same housing and management conditions. They were housed in standard single horse boxes (3 × 3 m) in visual contact with other conspecifics. They were fed twice a day, morning and afternoon, with hay and concentrate. Water was provided ad libitum by automatic drinkers. During the observation period, all horses were managed avoiding any changes in terms of workload, housing, rations, and daily routine. The owners were asked to fill out a questionnaire including information on the horse's characteristics and history as well as on the physical and social environment of the horse. Questions touched on home environment, management and feeding, and horse's use and exercise. Other specific questions regarded the medical history and development and presence of cribbing or other behaviour problems.

**Figure 1:**
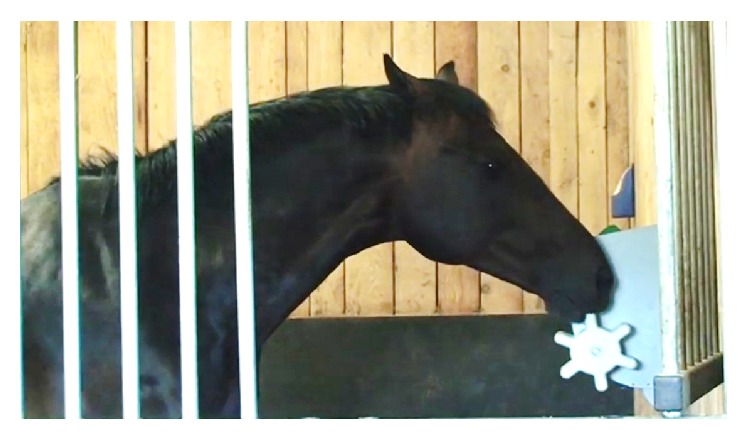
A horse approaches the dispenser wheel.

The design was a cross-over case-control study. In a preliminary phase, the horses were trained to use the dispenser Quaryka (Figure 1) that delivered small quantities of concentrate when a wheel was activated by the horse's mouth.

The training included four main steps lasting approximately two hours overall.

1. *Approach to the Dispenser*. The horse's attention was directed to the wheel by placing some concentrate feed on its spokes.
2. *Habituation to the Wheel Rotation*. When the horse approached the wheel with the muzzle, the wheel was manually rotated, so that the concentrate fell into the manger (this process allowed the animal to associate the movement of the wheel with the availability of food).
3. *Reward-Dependent Approach to the Wheel.* As soon as the horse touched the spokes with the lips, the experimenter turned the wheel.
4. *Autonomous Use of the Dispenser*. The horse approached the dispenser independently and the experimenter intervened only if the animal was distracted or did not apply enough force in turning the wheel. Each step was repeated several times, until the horse exhibited consistent learned behaviour.

After the preliminary phase, the observation period lasted 15 days divided into phases of five days each, for both groups of horses. During the first five days of testing (Pre-Quaryka), each subject was videorecorded in the box while fed concentrate in the usual location. During the next five days, the concentrate feed was distributed only through Quaryka (During-Quaryka) and, during the last five days, the dispenser was removed from the box and the horse was fed concentrate in the usual feeder (Post-Quaryka). During the study, the horses were continuously videotaped for one hour prior to and two hours after afternoon administration of the concentrate feed. Behaviour was recorded by a remotely operated video camera (Panasonic, HDC-SD99, Panasonic, Japan), mounted on a wall over the box and linked to a sequential switcher and time-lapse video recorder.

### 2.1. Data Analysis

The video recording analysis was carried out using dedicated software, the Solomon Coder (beta 12.09.04, copyright 2006–2008 by András Péter), customized with a specific behaviour configuration. An observer trained in animal behaviour and use of the software analysed all the videotapes. Behavioural categories are listed and described in Table 1. Owners' answers to the questionnaire were scored and reported.

### 2.2. Statistical Analysis

All statistical analyses were conducted using SPSS 21 (SPSS Inc., Chicago, USA). Differences were considered to be statistically significant if *P* ≤ 0.05. For each behaviour, mean duration and standard deviation were calculated. ANOVA (analysis of variance) was used to investigate potential differences in horse behaviour between groups or time periods.

## 3. Results and Discussion

Results from the questionnaire are summarized in Table 2. The majority of the horses (70%) were stabled on sawdust litter, while only 30% of them, of both groups, were stabled on wheat wood shavings. All boxes had an open window (overlooking indoor or outdoor) and the large majority of the subjects (80%) had daily access to grass paddocks, with shade and water available. Horses were fed with hay (a mean of 9 kg/day each) and concentrate (a mean of 3 kg/day each) to meet their specific energy requirements. All owners described their horse as “easy to manage” and “getting along with other horses.” All owners of* Cribbing horses* reported that the stereotypy was present at the time of purchase.

Most of the horses approached Quaryka with curiosity (60%), while the others (40%) showed signs of diffidence (no tactile exploration, standing alert) during the first 20 minutes.

The video analysis revealed that, during the entire observation period, none of the horses showed any of the behaviours related to frustration or fear described in Table 1 and none of the* Control horses* showed any displacement or stereotypic behaviour.

**Figure 2:**
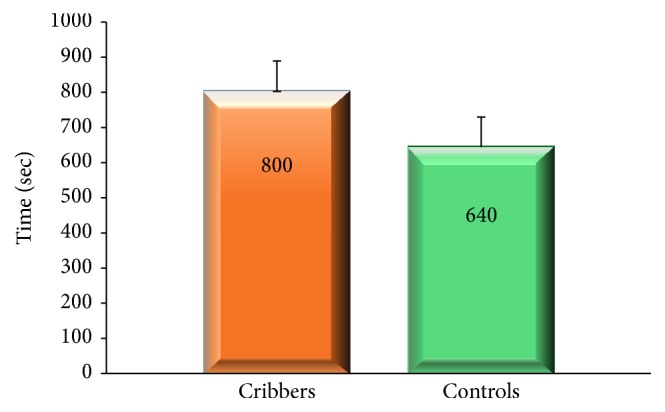
Time needed to finish the concentrate ration observed in Cribbing and Control horses during Pre-Quaryka phase of the study.

Figure 2 reports time to finish the ration recorded in horses during Pre-Quaryka.

Compared to* Control horses*,* Cribbing horses* tended to need a longer time to finish their concentrate, in agreement with findings of Clegg et al. [13] who found that cribbers and weavers took longer time than Control horses to fully consume their ration. This result can be explained considering that cribbers stereotype most frequently during and following the consumption of meals [16–20]. Only cribbers displayed lip playing during the observation time.

**Figure 3:**
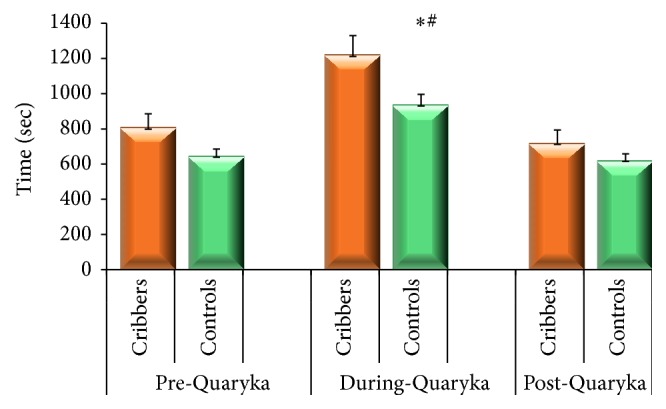
Time needed to finish the concentrate ration in Cribbing and Controls horses during the three phases of the study. ^*∗*^ *P* > 0.05 Cribbers versus Controls in During-Quaryka observation period. ^#^ *P* > 0.05 Cribbers and Control horses in During-Quaryka versus Pre-Quaryka observations.

Quaryka induced an increase in time needed to finish the ration in both groups of horses (Figure 3, *P* < 0.05). Also cribbers needed significantly more time to finish the ration than Control horses (*P* < 0.05). After Quaryka removal, horses in both groups showed a feeding behaviour similar to that expressed before the introduction of the dispenser (Figure 3).

**Figure 4:**
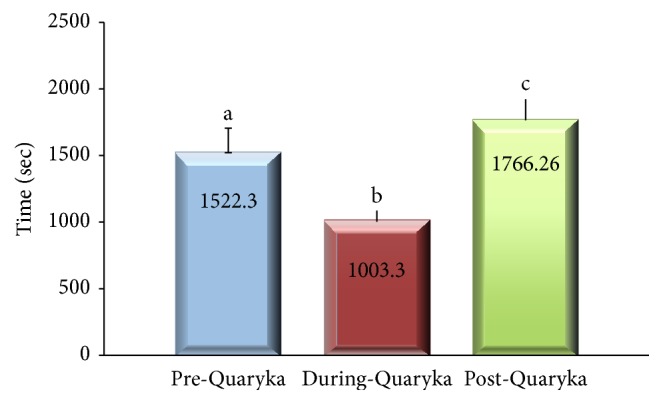
Cribbing group: cribbing behaviour observed during the three phases of the study. Bars with different superscripts differ significantly (*P* < 0.05).

Interestingly, cribbers showed a significant reduction of time spent cribbing (*P* < 0.05), indicating that horses' interaction with Quaryka induced lengthening of the time taken to finish the ration, in the absence of stereotyped behaviours associated with food consumption. After removal of the feed dispenser (Post-Quaryka), cribbing behaviour significantly increased compared to the previous phases (Figure 4). This result is compatible with a posttreatment rebound caused by a rise in the motivation to cribbing. Posttreatment rebound was observed in horses prevented from cribbing by the use of inhibitory systems [19]. This hypothesis should be considered with caution as in this study cribbing was never prevented. A possible alternative explanation may be related to the short exposure of the subjects to the dispenser not allowing a lasting effect on the stereotypic behaviour.

The effectiveness of Quaryka in reducing cribbing behaviour cannot be generalised due to the limited animal sample. It should be noted that, in all the horses included in this study, the use of Quaryka was associated with an increase of time needed to finish the ration. This may be particularly beneficial in horses fed high-energy diets and ingesting their food too quickly.

## Figures and Tables

**Table 1:** Behavioural categories and definitions.

Behavioural category	Definition	State/event
Time to finish the ration	Time taken by horse to finish the food ration	State
Time at the dispenser	Elapsed time at the dispenser, every time the horse shows interest in Quaryka (sniffs, spins the wheel, etc.)	State
Time to learn how to use Quaryka	Time spent learning to use the dispenser, until the horse understood the relationship between turning the wheel and the presence of concentrate in the manger	State
Latency to use Quaryka	Time needed by the horse to approach Quaryka the first time	State
Latency to use Quaryka after filling	Elapsed time between filling Quaryka and the horse turning the wheel to obtain the food for the first time	State
Time spent cribbing	Bout of cribbing	State
Standing alert	Rigid stance with the neck elevated and the head oriented toward the source of interest The ears are held stiffly upright and forward, and the nostrils may be slightly dilated	State
Fear	Fearful head posture and facial expression (increasing head distance from Quaryka, ears flattened and held back, and sclera visible)	State
Cribbing	Single crib-bite	Event
Lip playing	Part of tongue is shown and moved along the upper lip	Event

**Table 2:** Results of the questionnaire, filled out by the owners of the horses.

Horse #	# 1	# 2	# 3	# 4	# 5	# 6	# 7	# 8	# 9	# 10
Group	Control	Control	Control	Control	Control	Cribbing	Cribbing	Cribbing	Cribbing	Cribbing

Age (years)	6	13	17	17	17	18	10	11	6	20
Sex	Mare	Gelding	Stallion	Gelding	Mare	Gelding	Mare	Mare	Gelding	Gelding
Breed	English thoroughbred	Friesian	Sella Italiano	Selle Française	Hannover	Irish Horse	Sella Italiano	Dutch Warmblood	Selle Française	English thoroughbred
Height (meters)	1,62	1,60	1,52	1,65	1,65	1,62	1,58	1,67	1,63	1,61
Attitude (original)	Gallop	Equitation	Jumping	Jumping	Dressage	Dressage	Jumping	Jumping	Equitation	Jumping
Attitude (actual)	Equitation	Equitation	Jumping	Equitation	Dressage	Equitation	Equitation	Jumping	Equitation	Companion
Box size (square meters)	9	9	10,5	12	12	9	9	12	9	9
Bedding type	Wood shavings	Wood shavings	Wood shavings	Straw	Wood shavings	Straw	Straw	Wood shavings	Wood shavings	Wood shavings
Access to paddock	Daylight hours	Daylight hours	4 hours	4 hours	No routine access	Daylight hours	Daylight hours	No routine access	Daylight hours	Daylight hours
Cribbing since						Cribbing at purchase	Cribbing at purchase	Cribbing at purchase	Cribbing at purchase	Cribbing at purchase
Severity of cribbing behaviour						Mild	Severe	Mild	Mild	Severe
Initial reaction to Quaryka	Curiosity	Curiosity	Diffidence	Curiosity	Diffidence	Curiosity	Curiosity	Diffidence	Curiosity	Diffidence
